# Host cell virus entry mediated by Australian bat lyssavirus G envelope glycoprotein occurs through a clathrin-mediated endocytic pathway that requires actin and Rab5

**DOI:** 10.1186/1743-422X-11-40

**Published:** 2014-02-27

**Authors:** Dawn L Weir, Eric D Laing, Ina L Smith, Lin-Fa Wang, Christopher C Broder

**Affiliations:** 1 Department of Microbiology, Uniformed Services University, Bethesda, MD 20814, USA; 2 CSIRO Livestock Industries, Australian Animal Health Laboratory, Geelong, VIC, Australia; 3 Duke-NUS Graduate Medical School, Singapore 169857, Singapore

## Abstract

**Background:**

Australian bat lyssavirus (ABLV), a rhabdovirus of the genus *Lyssavirus* which circulates in both pteropid fruit bats and insectivorous bats in mainland Australia, has caused three fatal human infections, the most recent in February 2013, manifested as acute neurological disease indistinguishable from clinical rabies. Rhabdoviruses infect host cells through receptor-mediated endocytosis and subsequent pH-dependent fusion mediated by their single envelope glycoprotein (G), but the specific host factors and pathways involved in ABLV entry have not been determined.

**Methods:**

ABLV internalization into HEK293T cells was examined using maxGFP-encoding recombinant vesicular stomatitis viruses (rVSV) that express ABLV G glycoproteins. A combination of chemical and molecular approaches was used to investigate the contribution of different endocytic pathways to ABLV entry. Dominant negative Rab GTPases were used to identify the endosomal compartment utilized by ABLV to gain entry into the host cell cytosol.

**Results:**

Here we show that ABLV G-mediated entry into HEK293T cells was significantly inhibited by the dynamin-specific inhibitor dynasore, chlorpromazine, a drug that blocks clathrin-mediated endocytosis, and the actin depolymerizing drug latrunculin B. Over expression of dominant negative mutants of Eps15 and Rab5 also significantly reduced ABLV G-mediated entry into HEK293T cells. Chemical inhibitors of caveolae-dependent endocytosis and macropinocytosis and dominant negative mutants of Rab7 and Rab11 had no effect on ABLV entry.

**Conclusions:**

The predominant pathway utilized by ABLV for internalization into HEK293T cells is clathrin-and actin-dependent. The requirement of Rab5 for productive infection indicates that ABLV G-mediated fusion occurs within the early endosome compartment.

## Background

Australian bat lyssavirus (ABLV) is a rhabdovirus of the genus *Lyssavirus* endemic in Australian bat populations that is capable of causing a fatal neurological disease in humans indistinguishable from clinical rabies. There are two genetically distinct variants of ABLV: the *Pteropus* strain (ABLVp) which circulates in all four species of frugivorous flying foxes (genus *Pteropus*) present on mainland Australia, and the *Saccolaimus* strain (ABLVs) present in the insectivorous yellow-bellied sheathtail bat (genus *Saccolaimus*). ABLV has caused three fatal human infections, the most recent in February 2013, and each manifested as acute encephalitis; however, incubation periods were variable, ranging from approximately 5 weeks to over 2 years [1-3]. In May 2013, ABLV infected two horses, representing the first spillover of ABLV into a terrestrial species other than humans [4,5]. Although the number of ABLV spillover events have thus far been scarce and limited to only two terrestrial species, *in vitro* tropism studies indicate that the unknown ABLV receptor(s) is broadly conserved among mammals and suggests that terrestrial species other than humans and horses may be susceptible to ABLV infection [6].

ABLV is an enveloped, bullet-shaped, non-segmented, negative sense RNA virus belonging to the genus *Lyssavirus* of the family *Rhabdoviridae* within the order *Mononegavirales*. There are currently 12 species of lyssaviruses and 3 additional species that have not yet been classified [7]; ABLV is most closely related to classical rabies virus (RABV), the prototype member of the *Lyssavirus* genus. Lyssaviruses have a single envelope glycoprotein (G) that mediates all internalization steps from cell attachment to pH-dependent fusion with the host cell membrane [8]. The lyssavirus G glycoprotein is a key determinant of the neurotropic and neurovirulent properties of lyssaviruses [9].

Rhabdoviruses gain access to the host cell cytoplasm, the site of viral replication, by receptor-mediated endocytosis and subsequent low pH-dependent fusion [10,11]. Viruses internalized by receptor-mediated endocytosis predominantly use trafficking pathways mediated either by clathrin or macropinocytosis, but several alternative pathways have been reported (reviewed in [12,13]). Different viruses utilize different pathways for entry and the endocytic pathway taken by a given virus largely depends on the host receptor it interacts with and the size of the virus. Clathrin-mediated endocytosis (CME) is the most commonly utilized entry pathway for viruses small and intermediate in size [13] and is the only pathway reported to be utilized by different rhabdoviruses, including VSV, RABV, and infectious hematopoietic necrosis virus (IHNV) [14-16]. The initial virus-host cell receptor interaction induces de novo clathrin-coated pit (CCP) formation at the site of viral binding [17,18]. Both VSV and RABV were shown to internalize through partially coated clathrin pits that require actin to complete the internalization process [15,18]. Entry of IHNV was also shown to be actin-dependent [14]. After invagination, the CCP buds from the plasma membrane forming a clathrin-coated vesicle. The clathrin coat is then shed, enabling the uncoated vesicle to traffick to and fuse with the early endosome. The entry pathway of ABLV has not been examined; however, we recently demonstrated that disruption of lipid rafts via sequestration of cholesterol by methyl-β-cyclodextrin (MβCD) treatment significantly reduced ABLV G-mediated entry into HEK293T cells [6]. This finding is compatible with a clathrin- or caveolae -dependent (CavME) entry pathway for ABLV; acute cholesterol depletion with MβCD has been shown to inhibit CCP budding [19] and caveolae formation is strictly dependent on cholesterol [20].

Once internalized by endocytosis, the acidic environment of the endosome triggers the conformational changes in the G glycoprotein that lead to fusion of the viral and endosomal membranes and the subsequent release of the viral genome into the cytosol. For most enveloped viruses, membrane fusion occurs either in early endosomes (EEs) (pH 6.5-6.0) or late endosomes (LEs) (pH 6.0-5.0) (reviewed in [12]). Recycling endosomes (REs) are responsible for directing vesicular cargo back to the plasma membrane and have been shown to be involved in some virus budding pathways [21] and particle assembly pathways (reviewed in [22]). The use of dominant negative (DN) Rab GTPases specific for EEs, LEs, and REs, have been used extensively to determine virus exit points from the endosomal network [23-25]. Overexpression of the mutant Rab proteins in host cells elicits a dominant negative effect over the endogenous wild-type protein; thus, a virus that requires a specific endosome for trafficking and fusion will be adversely affected by the overexpression of a DN Rab protein specific for that endosome. Lyssaviruses require vacuolar acidification for G glycoprotein-mediated viral and endosomal membrane fusion [10], but few studies have examined the endosomal trafficking of these viruses. RABV G was shown to co-localize with Rab5a, a marker for EEs, but not Rab9, a marker for LEs; this same study demonstrated that internalization of an anti-RABV G antibody/RABV G complex was dependent on the presence of functional Rab5a suggesting that RABV G fuses with EEs [26]. Furthermore, the optimal pH for RABV G-mediated fusion is pH 5.8-6.0, which correlates with the pH of EEs [27]. The membrane fusion requirements for ABLV have not been investigated.

In the present study, we used fluorescent protein-encoding recombinant vesicular stomatitis viruses (rVSV) that express ABLV G envelope glycoproteins to examine the ABLV entry pathway using both chemical and molecular approaches. Results indicate that ABLV is internalized into HEK293T cells by a clathrin-dependent pathway that like VSV, IHNV, and RABV, is dependent upon actin. Moreover, we show that ABLV G-mediated entry is Rab5-dependent, but Rab7-independent, indicating that ABLV G-mediated fusion occurs within the early endosomal compartment.

## Results and discussion

### Dynamin is required for ABLV G-mediated viral entry

To examine the endocytic pathway utilized by ABLV for host cell internalization, we used maxGFP-encoding replication competent recombinant vesicular stomatitis viruses (rVSV) that express ABLV G glycoproteins [6]. This approach is advantageous over using WT ABLV because not only are rVSV-ABLV G viruses safer and easier to manipulate than WT ABLV, but the incorporation of GFP into the viral genome eliminates the need for traditional fluorescent antibody staining to detect infected cells. HEK293 cells, an immortalized cell line derived from primary human embryonic kidney cells in the late 1970s [28], were chosen as the cell culture model to investigate the ABLV entry pathway. In recent years, HEK293 cells been shown to express several genes that are typically found only in cells of neuronal origin, displaying neuronal gene expression patterns similar to those of early differentiating neurons or neuronal stem cells [29,30]. Moreover, a recent study demonstrated that HEK293 cells were just as sensitive as murine neuroblastoma cells for the rapid isolation of street RABV from brain tissue of suspected RABV infected animals [31]. Additionally, we previously demonstrated the high susceptibility of HEK293T cells to ABLV G-mediated viral infection [6]. The aforementioned neuronal characteristics of HEK293 cells combined with their ease of handling, robust growth rate, and amenability to transfection, make them an ideal model to study ABLV entry.

We first examined whether dynamin was required for ABLV entry. Dynamin, a GTPase that is responsible for the scission of endocytic vesicles from the plasma membrane [32], plays a critical role in several endocytic pathways including, but not limited to, CME [33], CavME [34], and some types of macropinocytosis [35,36]. In contrast, dynamin is dispensable for the macropinocytosis of vaccinia virus (VV) [37], the GPI-anchored protein-enriched endosomal compartment (GEEC) pathway [38], and a novel non-clathrin, non-caveolar entry pathway utilized by lymphocytic choriomeningitis virus (LCMV) [39]. Therefore, the effect of dynamin inhibition on viral entry serves as an initial criterion for endocytic pathway classification.

**Figure 1:**
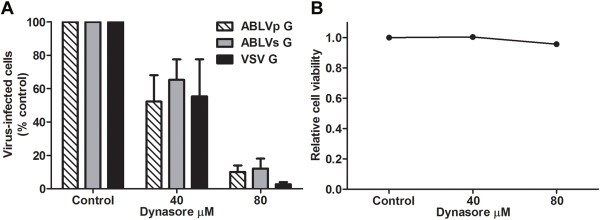
**Chemical inhibition of dynamin inhibits ABLV G-mediated viral entry into HEK293T cells. (A)** HEK293T cell monolayers were pretreated with dynasore diluted in OptiMEM® for 30 min at 37°C and were then infected with max-GFP encoding rVSV reporter viruses (MOI = 1). Cells were harvested 20 hrs post infection, fixed with 2% paraformaldehyde and then analyzed for GFP expression (indicative of productive infection). GFP positive cells were counted with a Nexcelom Vision automated cell counter with fluorescence detection and the percent of virus-infected cells was calculated by dividing the number of GFP positive cells by the total number of cells counted. Under these experimental conditions, a MOI of 1 yielded 60-70% virus-infected cells in untreated cells. Drug was maintained for the entire course of infection and its effect on cell viability **(B)** was determined by trypan blue staining. Reporter viruses that express VSV G were included as a positive control to assess dynasore activity. Results are expressed as percent virus-infected cells relative to that of untreated controls and represent 3 independent experiments; error bars are standard error of the mean (SEM).

HEK293T cells were pretreated with dynasore, a specific and potent inhibitor of dynamin [40], for 30 min at 37°C and then infected with rVSV-ABLV G reporter viruses; rVSV that expresses VSV G was included as a positive control. Dynasore treatment of HEK293T cells inhibited ABLV G-mediated viral entry in a dose dependent manner, with >80% inhibition at the highest concentration (Figure 1A). Impaired virus infection was not due to drug toxicity as cells were >90% viable at the end of the experiment (Figure 1B). These results indicate that ABLV G-mediated entry into HEK293T cells follows a dynamin-dependent pathway.

### Inhibition of clathrin-mediated endocytosis inhibits ABLV G-mediated viral entry

**Figure 2:**
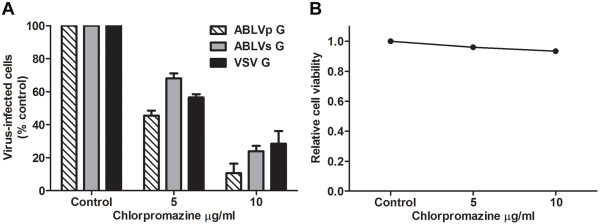
**Chemical inhibition of CME inhibits ABLV G-mediated viral entry into HEK293T cells. (A)** HEK293T monolayers were pretreated with chlorpromazine diluted in OptiMEM® for 30 min at 37°C. Cells were then infected with rVSV (MOI = 3) for 8 hrs and then analyzed as described in Figure 1. Under these conditions, a MOI of 3 yielded 50-60% virus-infected cells in untreated cells. Drug was maintained for the entire course of infection and its effect on cell viability **(B)** was determined by trypan blue staining. The rVSV-VSV G reporter virus was included as positive control. Results are expressed as percent virus-infected cells relative to that of controls and represent 3 independent experiments; error bars are SEM.

CME is the endocytic route most frequently utilized by small to intermediate sized viruses to gain entry into host cells [13]. Other rhabdoviruses such as VSV, IHNV, and RABV have all been reported to utilize CME for entry [14-16]. To assess whether clathrin is required for ABLV entry, we first employed chemical inhibition studies using chlorpromazine, a drug that inhibits CME by blocking the assembly of clathrin coated pits [41]. HEK293T cells were pretreated with chlorpromazine at the indicated concentrations for 30 min at 37°C prior to infection with rVSV-ABLV G reporter viruses in the presence of drug; rVSV-VSV G was included as a positive control. To avoid cell toxicity associated with long-term exposure to chlorpromazine, cells were harvested 8 hrs post infection and analyzed for GFP expression. Chlorpromazine inhibited ABLV G-mediated entry in a dose-dependent manner, with >70% inhibition at the highest concentration (Figure 2A). To monitor potential toxic effects of chlorpromazine treatment, cell viability was assessed in parallel. Under these experimental conditions, no toxicity was noted; greater than 90% of cells treated with the highest concentration were viable at the end of the experiment (Figure 2B).

**Figure 3:**
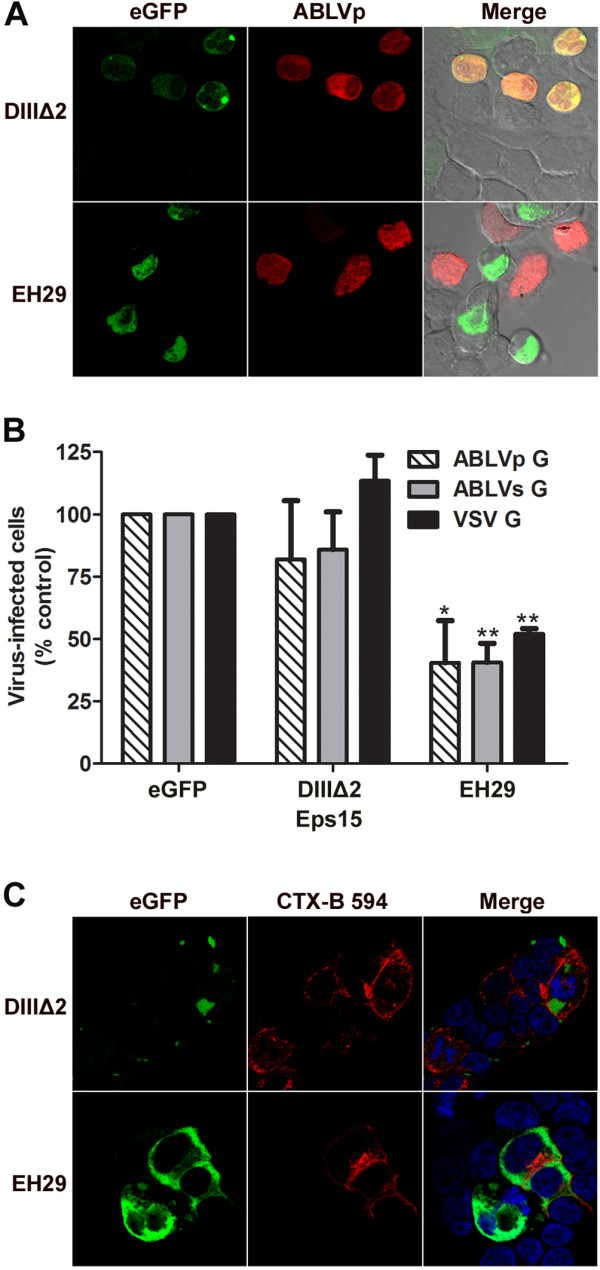
**Inhibition of CME with a DN form of Eps15 significantly reduces ABLV G-mediated viral entry into HEK293T cells.** HEK293T cells transfected with an eGFP control plasmid or eGFP-tagged Eps15 mutants were infected with VSVΔG-ABLV G* or –VSV G*-RFP pseudoviruses at a MOI = 2 for 20 hrs and then analyzed for RFP expression as described in Figure 1. Under these conditions, a MOI of 2 yielded 25-30% virus-infected cells among cells transfected with the eGFP control plasmid. DIIIΔ2, a mutant of Eps15 that does not affect clathrin coated pit formation; EH29, DN Eps15 mutant. **(A)** Representative microscopic fields of cells transfected with each Eps15 construct and infected with VSVΔG-ABLVp G*- RFP pseudovirus. Similar results were obtained for ABLVs G-expressing pseudovirus (not shown). **(B)** Quantitation of virus-infected cells. A minimum of 1000 cells were counted for each sample per experiment. VSVΔG- VSV G* was included as positive control. Results are expressed as percent virus-infected cells relative to that of controls and represent 3 independent experiments; error bars are SEM. Significance of effect on virus infection was determined using Student’s *t*-test. **, p < 0.0005; *, p < 0.005. **(C)** Cholera toxin B (CTX-B) subunit uptake into HEK293T cells is not inhibited by EH29. To verify the specificity of EH29 to inhibit CME, 18 hrs post transfection HEK293T cell monolayers grown on 12 mm coverslips were incubated with Alexa Fluor 594-labeled CTX-B (10 μg/ml) for 1 hr at 37°C. Cells were then washed twice with PBS, fixed and imaged. Images were taken by confocal microscopy with a mid z-section shown. Nuclei were stained with DAPI (4′,6-diamidino-2-phenylindole, dihydrochloride).

To confirm CME as an entry pathway for ABLV, we next examined the effect of the expression of a dominant negative (DN) mutant (EH29) of the CME effector protein Eps15. The EH29 mutant blocks CME by preventing clathrin-coated pit formation and does not affect non-clathrin pathways; Eps15 mutant DIIIΔ2, which has no effect on clathrin-coated pit assembly, was included as a negative control for the inhibition of CME [42-44]. HEK293T cells were transfected with plasmids encoding eGFP alone or eGFP-tagged Eps15 mutants for 18 hrs; transfection rates, as measured by eGFP expression, consistently reached 50-60% (data not shown). Cells were then infected with VSVΔG-RFP pseudoviruses that express ABLV G or VSV G at a MOI = 2. After 20 hrs the cells were harvested and analyzed for RFP expression. Representative microscopic fields of cells transfected with each Eps15 construct and infected with VSVΔG-ABLVp G*- RFP pseudovirus are shown in Figure 3A. Similar results were obtained for ABLVs G-expressing pseudovirus (not shown). As expected, compared to the eGFP control, DIIIΔ2 did not interfere with ABLV entry (Figure 3B); however, ABLV G-mediated viral entry was significantly inhibited by EH29 (60%) compared to eGFP controls (Figure 3B). The uptake of cholera toxin B subunit (CTX-B), which is endocytosed by CavME [45], was not inhibited by EH29, demonstrating the specificity of EH29 in inhibiting CME (Figure 3C). Taken together, these data suggest that ABLV G-mediated viral entry into HEK293T cells occurs through a clathrin-dependent pathway.

### Caveolar-dependent endocytosis and macropinocytosis are not involved in ABLV G-mediated viral entry

**Figure 4:**
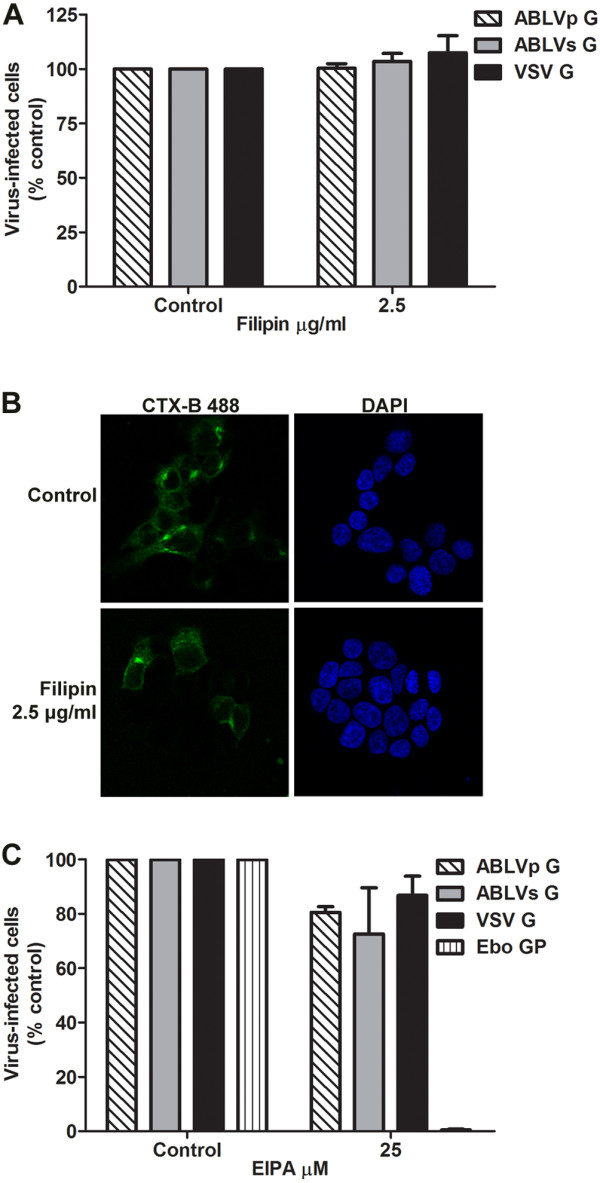
**CavME endocytosis and macropinocytosis are not required for ABLV G-mediated viral entry. (A)** Chemical inhibition of CavME. HEK293T cell monolayers were pretreated with filipin diluted in OptiMEM® for 1 hr at 37°C. Cells were then infected with rVSV (MOI = 1) for 20 hrs and analyzed as described in Figure 1. Drug was maintained for the entire course of infection. **(B)** Cholera toxin B (CTX-B) subunit uptake is inhibited by filipin. Following pretreatment with filipin as described in **(A)**, HEK293T cell monolayers grown on 12 mm coverslips were incubated with Alexa Fluor 488-labeled CTX-B (10 μg/ml) for 1 hr at 37°C. Cells were then washed twice with PBS, fixed and imaged. Images were taken by confocal microscopy with a mid z-section shown. Nuclei were stained with DAPI, (4′,6-diamidino-2-phenylindole, dihydrochloride). **(C)** Chemical inhibition of macropinocytosis. HEK293T cell monolayers were pretreated with EIPA diluted in OptiMEM® for 1 hr at 37°C. Cells were then infected with rVSV (MOI = 1) for 20 hrs and analyzed as described in Figure 1. rVSV encoding VSV G (MOI = 1) or EboGP (MOI = 15) were included as negative and positive controls, respectively, to assess EIPA activity. Drug was maintained for the entire course of infection. For **(A)** and **(C)** results are expressed as percent virus-infected cells relative to that of untreated controls and represent 3 independent experiments; error bars are SEM. Under these experimental conditions, the chosen MOIs yielded 60-70% virus-infected cells in untreated controls. EIPA, 5-(N-ethyl-N-isopropyl) amiloride.

Some viruses, such as Ebola virus [46,47], influenza A [48,49], and reovirus [50], have been reported to utilize more than one type of endocytic pathway to gain entry into host cells. Additionally, we previously showed that the disruption of lipid rafts through cholesterol depletion significantly reduced ABLV G-mediated entry into HEK293T cells [6], a finding compatible with a clathrin- or caveolae-dependent entry pathway. To test the possible contribution of pathways other than CME to ABLV G-mediated viral entry, HEK293T cells were treated with the chemical inhibitors filipin and EIPA (5-(N-ethyl-N-isopropyl amiloride)) to inhibit CavME and macropinocytosis, respectively. A recombinant VSV reporter virus expressing VSV G and fluorescently labeled CTX-B were included as a negative and positive control, respectively for CavME [45]; rVSV-EboGP reporter virus was included as a positive control for macropinocytosis [47]. Pretreatment of HEK293T cells with filipin, which disrupts CavME by specifically binding to cholesterol abundant in caveolae [51,52], did not inhibit ABLV G-mediated entry (Figure 4A), but did significantly decrease the uptake of CTX-B into HEK293T cells (Figure 4B). Similarly, pretreatment of HEK293T cells with EIPA, a potent inhibitor of Na+/H+ exchanger activity required for macropinosome formation [53,54], had no effect on ABLV G-mediated entry but, as previously reported, drastically inhibited Ebo GP-mediated viral entry (>98%) (Figure 4C) [47]. Taken together, these data indicate that CavME and macropinocytosis do not contribute to ABLV G-mediated entry into HEK293T cells. This is in agreement with previous studies that show that CavME and macropinocytosis do not play a role in the entry of other rhabdoviruses [14,15,47].

### Actin polymerization is required for ABLV G-mediated viral entry

**Figure 5:**
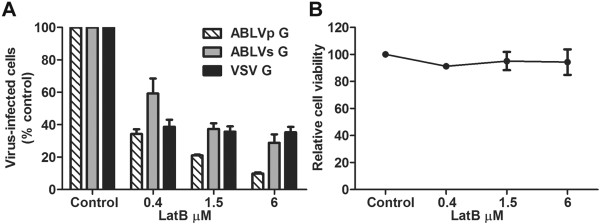
**Actin is required for ABLV G-mediated viral entry. (A)** HEK293T cell monolayers were pretreated with latrunculin B (LatB) diluted in OptiMEM® for 1 hr at 37°C. Cells were then infected with max-GFP encoding rVSV reporter viruses (MOI = 3). Under these conditions, a MOI of 3 yielded at least 50% virus-infected cells in untreated controls. Cells were harvested 8 hrs post infection and analyzed as described in Figure 1. Drug was maintained for the entire course of infection and its effect on cell viability **(B)** was determined by trypan blue staining. Reporter viruses that express VSV G were included as a positive control to assess LatB activity. Results are expressed as percent virus-infected cells relative to that of untreated controls and represent 3 independent experiments; error bars are standard error of the mean (SEM).

Recent studies have revealed that the rhabdoviruses VSV, RABV, and IHNV are internalized through clathrin-dependent pathways that are also actin-dependent [14,15,18]. To determine whether ABLV entry is also dependent upon actin, HEK293T cells were pretreated with the actin depolymerizing drug latrunculin B (LatB) at the indicated concentrations for 1 hr at 37°C prior to infection with rVSV-ABLV G reporter viruses in the presence of drug; rVSV-VSV G was included as a positive control. To avoid cell toxicity associated with long-term exposure to LatB, cells were harvested 8 hrs post infection and analyzed for GFP expression. LatB significantly inhibited ABLV G-mediated entry at all concentrations tested with greater than 65% and 90% inhibition of ABLVs G- and ABLVp G-mediated entry, respectively, at the highest concentration tested (Figure 5A). Entry inhibition was not due to drug toxicity as cell viability remained greater than 85% at the highest dose of LatB (Figure 5B).

Actin is not required for the clathrin-mediated uptake of transferrin, the classical ligand used to study CME [15,55]; however, it is required for CME of large cargos such as viruses and bacteria [15,18,56]. VSV and RABV in particular have been shown to be internalized by partially coated pits that require actin polymerization for envelopment; this dependence on actin is dictated by the size of the particle, as truncated defective-interfering particles of VSV do not require actin [15,18,57]. Thus, given the shared particle morphology of all rhabdoviruses, it is not surprising that ABLV entry is also actin-dependent. The mechanism by which cell surface bound viral particles induce actin recruitment to the CCPs is not completely understood. However, recent studies have demonstrated that plasma membrane tension induces the actin dependence of clathrin coat assembly; in the case of tense or rigid membranes, actin polymerization may be required to produce sufficient force to complete membrane deformation into a coated pit prior to the recruitment of dynamin and subsequent vesicle budding [58,59]. It has been postulated that upon cell surface binding, the viral particles themselves induce membrane tension, thus leading to the recruitment of actin to the forming clathrin-coated vesicle [15,18,57,58].

### Rab5, but not Rab7 or Rab11, is required for ABLV G-mediated viral entry

The above data indicate that ABLV G-mediated viral entry into HEK293T cells occurs through a clathrin- and actin-dependent pathway. To investigate the subsequent trafficking route to the site of cytosol penetration, we examined the roles in ABLV entry of the small GTPases Rab5, Rab7, and Rab11, which are involved in vesicular trafficking to early, late, and recycling endosomes, respectively [12]. HEK293T cells were transfected with RFP-tagged dominant negative (DN) forms of Rab5 (S34N) [60], Rab7 (T22N) [61], and Rab11 (S25N) [62], which prevent the fusion of endocytic vesicles with early endosomes, prevent movement from early to late endosomes, and prevent movement from early to recycling endosomes, respectively. Transfected cells were infected with rVSV-ABLV G 18–20 hrs post-transfection. Cells were fixed 8 hrs later and analyzed for GFP expression. Reporter viruses that express VSV G and Ebo GP were included as positive controls for Rab5 and Rab7, respectively [25,47].

**Figure 6:**
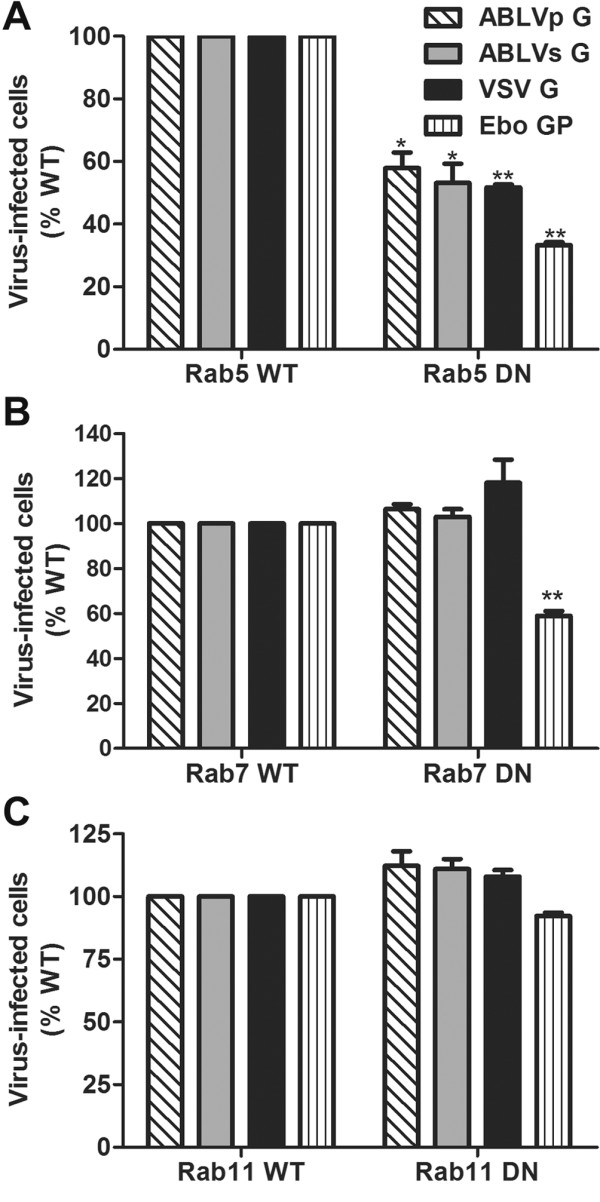
**Effect of dominant negative Rab GTPases on ABLV G-mediated viral entry.** HEK293T cells expressing dsRed-tagged WT and DN Rab5, Rab7, and Rab11were infected with maxGFP encoding rVSV that express ABLVp G, ABLVs G, or VSV G at a MOI = 3 for 8 hrs or with rVSV that expresses EboGP at a MOI = 15 for 20 hrs and then analyzed as described in Figure 1. Infection of all cells was assessed. Under these conditions, the chosen MOIs yielded 40-50% virus-infected cells in controls. Results are expressed as percent virus-infected cells relative to that of WT Rab controls and represent 3 independent experiments; error bars are SEM. **(A)** Rab5. **(B)** Rab7. **(C)** Rab11. **, p < 0.0001; *, p < 0.005.

As shown in Figure 6A, expression of the Rab5 DN significantly reduced (p < 0.005) ABLV G-mediated viral entry compared to Rab5 WT, with greater than 40% inhibition for both ABLV variants (Figure 6A); as previously reported, both VSV G- and Ebo GP-mediated entry was significantly inhibited by Rab5 DN (p < 0.0001) (Figure 6A) [23,25,47]. Expression of Rab7 DN and Rab11 DN did not inhibit ABLV G-mediated viral entry, but rather enhanced infection compared to Rab7 and 11 WT plasmids (Figure 6B-C); however, the increase in infectivity was not statistically significant. Expression of the Rab7 DN was sufficient to disrupt trafficking to late endosomes as Ebo GP-mediated viral entry was significantly inhibited (p < 0.0001) (Figure 6B) [47]. In agreement with previous studies, VSV G-mediated viral entry was not inhibited by DN mutants of Rab7 and 11 (Figure 6B-C) [25]. Similar to results for ABLV G-mediated infection, the apparent increased VSV infectivity of cells expressing DN Rab7 and 11 mutants compared to cells expressing WT Rabs was not statistically significant.

Rab11 is required for the transport of cargo from the Trans-Golgi network to the plasma membrane [63] and has been shown to play roles in virus assembly/budding [21,22]; however, consistent with our findings, there have been no reports of Rab11 playing a role in viral entry. Nevertheless, both VSV G and RABV G have been shown to colocalize with Rab11 [26,63] and the expression of the Rab11 DN has previously been shown to lead to accumulation of VSV G in the Golgi [63]. These studies suggest that Rab11 may play a post-entry role in trafficking of newly synthesized G from the Golgi to the plasma membrane. It is possible that a similar role for Rab11 in ABLV G trafficking exists, however this has not been investigated. Collectively, these data indicate that ABLV G-mediated fusion of viral and host cell membranes is triggered upon delivery to early endosomes and that productive ABLV infection does not require transfer to late or recycling endosomes. Further supporting this argument, and similar to that of RABV G [27], ABLV G-mediated fusion occurs at pH 6.0-6.2 with optimal fusion between pH 5.7–5.9 (data not shown), which correlates with the pH of EEs [12].

## Conclusions

In summary, we have used both chemical and molecular approaches to examine the entry pathway utilized by ABLV for internalization into HEK293T cells. The results presented here reveal that ABLV G-mediated viral entry primarily follows a clathrin-dependent pathway that, similar to other rhabdoviruses including RABV, requires actin for productive infection. Moreover, we show that productive ABLV infection is dependent upon Rab5, but not Rab7 or Rab11, indicating that the mildly acidic environment of the early endosome is sufficient to trigger ABLV G-mediated viral and endosomal membrane fusion and subsequent release of the viral genome into the cytosol.

The identification of specific entry requirements for a given virus is an important first step towards the development of antivirals capable of blocking infection. This study is the first to identify specific host factors required for ABLV entry. Our ongoing studies are aimed at identifying additional host factors required for ABLV infection using a high-throughput siRNA screening approach. Based on the results presented here, which indicate that ABLV follows a similar entry pathway as RABV [15], it is possible that the identification of host factors required for ABLV infection will provide potential targets for the development of therapeutics with the potential of broadspectrum activity against other lyssavirus species.

## Methods

### Cells and viruses

HEK293T cells were provided by Gerald Quinnan (Uniformed Services University) and were maintained in Dulbecco’s modified Eagle’s medium (DMEM; Quality Biologicals, Gaithersburg, MD) supplemented with 10% cosmic calf serum (CCS) (Hyclone, Logan, UT) and 2 mM L-glutamine (DMEM-10). Recombinant maxGFP expressing vesicular stomatitis viruses (rVSV) that express ABLV G, Ebola Zaire GP, and VSV (Indiana) G glycoproteins have been previously described [6]. VSVΔG-G*-RFP pseudovirus stocks were kindly provided by Michael Whitt (University of Tennessee). VSVΔG pseudoviruses complemented with ABLV G glycoproteins were prepared by transfecting HEK293T cells with expression plasmids that express ABLVs G and ABLVp G [6]. Twenty four hours after transfection, cells were infected with VSVΔG-G*-RFP at a multiplicity of infection (MOI) of 1 for 1 hr at 37°C. The cells were then washed three times with phosphate buffered saline (PBS) and DMEM-10 was added. After 24 hrs, culture supernatant was collected and cell debris was removed by centrifugation at 2,600 rpm for 10 min. Clarified supernatant was layered on top of 20% sucrose in TNE buffer (10 mM Tris, 135 mM NaCl, 2 mM EDTA) and centrifuged at 27,000 rpm for 2 hrs. Pseudovirus pellets were resuspended in 10% sucrose/TNE buffer. Viral titers were determined by adding serial 10-fold dilutions of virus to HEK293T cells grown on 96-well plates. At 24 hrs post-infection, fluorescent cells were counted under a fluorescent microscope and calculated as infectious units/ml (IU/ml).

### Drug treatments and cell infection assays

All chemical inhibitors were purchased from Sigma (St. Louis, MO). Stock solutions were prepared in water (chlorpromazine) or DMSO (dynasore, filipin, EIPA [5-(N-ethyl-N-isopropyl) amiloride], and latrunculin B) and stored, as per manufacturer’s recommendation. HEK293T cells were pretreated for 30 min in the presence of dynasore or chlorpromazine or for 1 hr in the presence of filipin, EIPA, or latrunculin B (LatB) at the indicated concentrations and then infected with rVSV viruses (rVSV-ABLV G and -VSV G, MOI = 1 or 3 for 20 hr and 8 hr infections, respectively; rVSV-EboGP, MOI = 15). Under these experimental conditions, the above MOIs resulted in 60-70% or 50-60% virus-infected cells in untreated controls for 20 hr and 8 hr infections, respectively. All drugs and viruses were diluted in OptiMEM® (Invitrogen, Carlsbad, CA) and drugs were maintained for the entire course of infection. Cells were harvested 20 hrs post infection (p.i.) for dynasore, filipin, and EIPA treatments, and 8 hrs p.i. for chlorpromazine and latrunculin B treatments. Single-cell preparations were made and fixed (2% paraformaldehyde (PFA) in PBS) and GFP expression, indicative of productive infection, was analyzed by a Nexcelom Vision automated cellometer (Nexcelom Bioscience LLC., Lawrence, MA) capable of fluorescence detection. The percent of infected cells was calculated by dividing the number of GFP positive cells by the total number of cells and multiplying by 100. At least 1000 cells were counted per sample for each experiment. Results are expressed as percent virus-infected cells relative to that of untreated controls and represent 3 independent experiments; error bars are standard error of the mean (SEM). Cell viability of drug treated cells was determined by trypan blue staining.

### Analysis of dominant negative mutants of Eps15, Rab5, Rab 7, and Rab11

Eps15 control (DIIIΔ2) and dominant negative (DN) (EH29) eGFP-tagged plasmids were kindly provided by Robert Davey (Texas Biomedical Research Institute). A plasmid expressing eGFP (pEGFP-C1; Clonetech, Mountain View, CA) was used as an additional control in Eps15 experiments. DsRed-tagged Rab7 wild-type (WT) (plasmid #12661) and DN (T22N; plasmid #12662), DsRed-tagged Rab11 WT (plasmid #12679) and DN (S25N; plasmid #12680) [64], and mRFP-tagged Rab5 WT (plasmid #14437) [65] were purchased from Addgene, Cambridge, MA. RFP-tagged Rab5 DN (S34N) was generated by site-directed mutagenesis of Rab5 WT using a QuikChange II site-directed mutagenesis kit (Stratagene, Cedar Creek, TX). HEK293T cells grown to 50% confluence in 24-well tissue culture plates were transfected with plasmids using Lipofectamine LTX (Invitrogen) according to the manufacturer’s protocol. Eighteen to twenty hours post transfection, cells were infected with VSVΔG-ABLV G*-RFP at a MOI = 2 for 20 hrs (Eps15 transfected cells) or with rVSV-ABLV G-GFP at a MOI = 3 for 8 hrs (Rab transfected cells). VSVΔG-VSV G*-RFP (MOI = 2) was included as a positive control for Eps15 DN functionality. Recombinant VSV-GFP reporter viruses that express VSV G (MOI = 3, 8 hr infection) and EboGP (MOI = 15, 20 hr infection) were included as positive controls for Rab5 DN and Rab7 DN functionality, respectively. Under these experimental conditions, the chosen MOIs yielded 25-30% and 40-50% virus-infected cells in controls for the Eps15 and Rab experiments, respectively. Cells were harvested and analyzed for RFP or GFP expression (Eps15 and Rab experiments, respectively) using a Nexcelom Vision cell counter as described above. At least 1000 cells were counted per sample per experiment. Results are expressed as percent virus-infected cells relative to that of controls and represent 3 independent experiments; error bars are SEM. For confocal imaging, HEK293T cells grown to 30% confluence on 12 mm coverslips were transfected and infected as described above. Cells were fixed with 4% PFA and then the coverslips mounted on slides using SouthernBiotech™ Dapi-Fluoromount-G™ clear mounting media and imaged by confocal microscopy.

### Cholera toxin B subunit uptake

HEK293T cells grown on 12 mm coverslips in 24-well tissue culture plates were pretreated with filipin or transfected with Eps15 plasmids as described above and then incubated with Alexa Fluor (AF) 488- or AF 594-conjugated cholera toxin B subunit (Invitrogen) (10 μg/ml) diluted in OptiMEM® for 1 hr at 37°C. Cells were washed twice with PBS and fixed with 4% PFA. The coverslips were mounted on slides using SouthernBiotech™ Dapi-Fluoromount-G™ clear mounting media and imaged by confocal microscopy.

### Confocal microscopy

Confocal images were obtained using a 63× oil objective, a Zeiss 710 NLO microscope, and Zen software. LSM files were exported to Adobe Photoshop for cropping and contrast adjustments.

### Statistical analysis

Student’s *t*-test was used to evaluate the statistical significance levels of the data, with p < 0.05 indicating statistical significance.

## Competing interests

The authors declare that they have no competing interests.

## Authors’ contributions

DLW designed and performed experiments, analyzed data and wrote the manuscript. EDL performed the confocal microscopy experiments. ILS and LFW provided important reagents and sequence information. CCB contributed to the design of experiments, provided overall supervision and financial support and edited the manuscript. All authors read and approved the final manuscript.
